# Renal Biopsy in Type 2 Diabetic Patients

**DOI:** 10.3390/jcm4050998

**Published:** 2015-05-18

**Authors:** Eugenia Espinel, Irene Agraz, Meritxell Ibernon, Natalia Ramos, Joan Fort, Daniel Serón

**Affiliations:** Nephrology Department, Hospital Universitari Vall d’Hebron, Autonomous University of Barcelona, Passeig de la Vall d’Hebron 119-129, 08035 Barcelona, Spain; E-Mails: eespinel@vhebron.net (E.E.); iagraz@vhebron.net (I.A.); mibernon@vhebron.net (M.I.); nramos@vhebron.net (N.R.); jfort@vhebron.net (J.F.)

**Keywords:** diabetic nephropathy, type 2 diabetes, kidney biopsy, histology

## Abstract

The majority of diabetic patients with renal involvement are not biopsied. Studies evaluating histological findings in renal biopsies performed in diabetic patients have shown that approximately one third of the cases will show pure diabetic nephropathy, one third a non-diabetic condition and another third will show diabetic nephropathy with a superimposed disease. Early diagnosis of treatable non-diabetic diseases in diabetic patients is important to ameliorate renal prognosis. The publication of the International Consensus Document for the classification of type 1 and type 2 diabetes has provided common criteria for the classification of diabetic nephropathy and its utility to stratify risk for renal failure has already been demonstrated in different retrospective studies. The availability of new drugs with the potential to modify the natural history of diabetic nephropathy has raised the question whether renal biopsies may allow a better design of clinical trials aimed to delay the progression of chronic kidney disease in diabetic patients.

## 1. Introduction

Diabetes mellitus (DM) represents one of the most important health problems worldwide. Over the last years, the global prevalence of type 2 diabetes mellitus (T2DM) has reached epidemic proportions fuelled by the global rise in the prevalence of obesity and unhealthy lifestyles. The World Health Organization foresees an increase in the global prevalence of DM from 2.8% in year 2000 to 4.4% in year 2030. This estimation represents that there will be 366 million adults with diabetes in 2030 [1]. In Spain, between 6% and 10% of the adult population suffers from DM and this percentage is as high as 12% in The Canary Islands [2]. Between 6.3% and 7.4% of the total budget of the Spanish National Health Service is spent in diabetes care, which represents an annual cost of 1.290 € to 1.476 € per patient [3].

Diabetes Mellitus is the fifth-leading cause of death worldwide and is associated with an increased risk of cardiovascular disease [4]. Chronic kidney disease (CKD) is a common complication in diabetic patients and further contributes to increased mortality and cardiovascular disease. The prevalence of CKD defined as an estimated glomerular filtration rate (e-GFR) <60 mL/min/1.73 m^2^ and/or the presence of microalbuminuria in adults with T2DM was 45% and raised to 61% in patients >65 years according to the NHANES report [5]. Furthermore, diabetic nephropathy is the most common cause of end-stage renal disease in Western Societies, accounting for approximately 45% of patients on renal replacement therapy. Moreover, patient survival in diabetics on maintenance dialysis is lower than in non-diabetic patients with end-stage renal disease due to other renal diseases [6].

## 2. Clinical Diagnosis of Diabetic Kidney Disease

The first clinical sign suggesting renal involvement due to diabetes is hyperfiltration characterized by increased glomerular filtration rate over 120 mL/min/1.73 m^2^, which is followed by the onset of microalbuminuria (albumin excretion >30 mg/g creatinine).

The natural history of the renal involvement in diabetes is better characterized in patients with type I diabetes mellitus (T1DM) since the beginning of diabetes is precisely known. It has been observed that microalbuminuria in patients with T1DM rarely appears within the first five years. Without specific intervention, 80% of type I diabetic patients that develop microalbuminuria will evolve to macroalbuminuria (albumin excretion >300 mg/g creatinine) at an average time of 10–15 years. During this period of time, hypertension will also appear. Once macroalbuminuria is present, the glomerular filtration rate decreases progressively at a variable rate, ranging between 2–20 mL/min/year. Approximately 50% of patients with T1DM and macroalbuminuria will progress to end stage renal disease in a period of 10 years and 75% in a period of 20 years [7].

**Figure 1:**
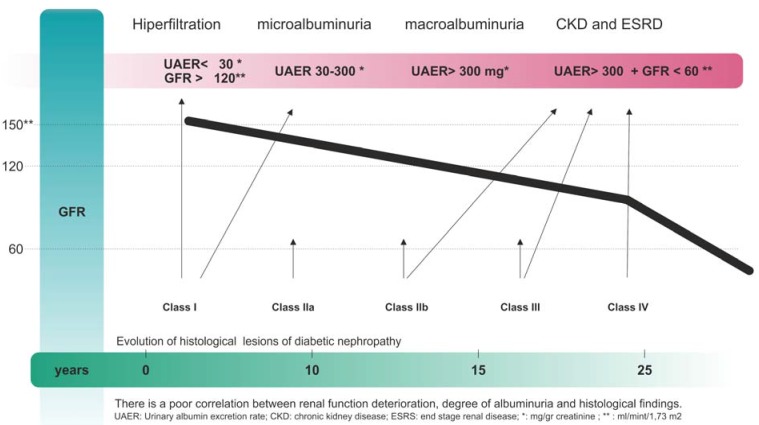
Natural history of diabetic nephropathy. There is a poor correlation between renal function deterioration, degree of albuminuria and histological findings. UAER: Urinary albumin excretion rate; CKD: chronic kidney disease; ESRS: end stage renal disease; * mg/gr creatinine; ** mL/mint/1.73 m^2^.

The natural history of diabetic nephropathy is less well established in T2DM since alterations of glucose metabolism are indolent and the diagnosis of diabetes is usually established many years afterwards. A proportion of patients with T2DM already display micro or even macroalbuminuria at the time of diagnosis. Without specific intervention, 20% to 40% of patients with T2DM presenting microalbuminuria are going to progress to macroalbuminuria. However, 20 years after the beginning of macroalbuminuria, only 20% of patients will progress to end stage renal disease. In Figure 1, the natural history of diabetic nephropathy is summarized [7].

**Figure 2:**
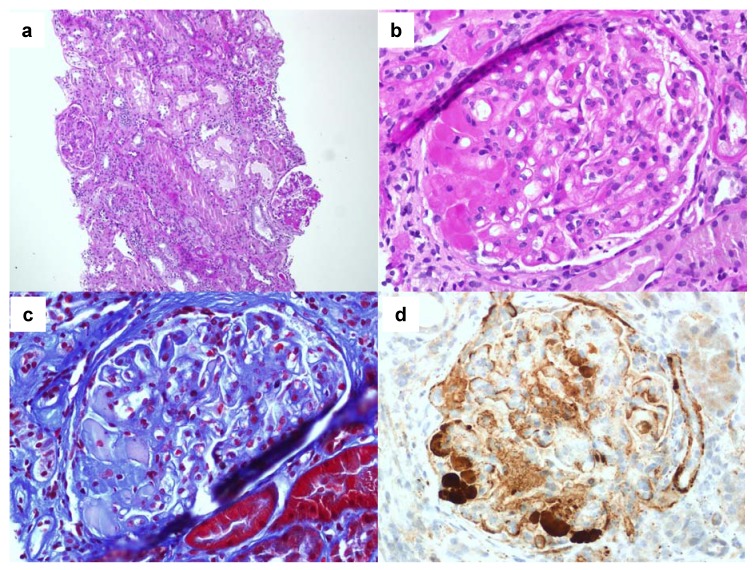
A patient with nephrotic syndrome, type 2 diabetes and hepatitis C virus associated crioglobulinemia. (**a**) Hematoxilyn-eosin at 100× magnification showing a glomerulus with mesangial expansion and another with glomerular thombi; (**b**) periodic acid Schiff (PAS) stain at 400× magnification of the glomerulus showing thrombi; (**c**) same glomerular section at 400× magnification stained with Masson’s trichrome (**d**) immunohistochemistry showing subendothelial C3 deposition in the glomerular lumen.

Diabetic glomerulopathy is the characteristic lesion of patients with diabetic nephropathy. However, only a small proportion of diabetic patients are biopsied. Accordingly, the term diabetic kidney disease (DKD) is the preferred one to refer to patients in whom diabetic nephropathy is suspected on clinical grounds [8]. The term diabetic nephropathy should only be employed in diabetic patients with histological confirmation of renal involvement due to diabetes [9]. Thus, the term DKD represents a probability for the diagnosis of diabetic nephropathy and the presence of renal insufficiency and/or albuminuria in diabetic patients can be due to other renal diseases different from diabetic nephropathy. In a study reviewing 620 biopsies made in patients with diabetes in 2011, Sharma *et al.*, showed that among 2642 native kidney biopsies, 37% of patients showed pure diabetic nephropathy, 36% showed a non-diabetic renal disease and in 27% of patients diabetic nephropathy was associated with a non-diabetic renal disease [10]. In other studies evaluating the renal diagnosis in diabetic patients, similar results have been reported [11]. In Figure 2, a diabetic patient with hepatitis C virus, and nephrotic syndrome showing intraglomerular thrombi associated with crioglobulinemia is shown.

The probability to suffer from diabetic nephropathy is high in patients with albuminuria and diabetic retinopathy [12]. Absence of diabetic retinopathy even in the presence of macroalbuminuria is associated with a high probability of non-diabetic renal disease. Approximately 30% of diabetic patients with macroalbuminuria without diabetic retinopathy will show normal histology or renal diseases different from diabetic nephropathy [13,14]. Thus, the absence of retinopathy is considered an indication for renal biopsy in albuminuric patients. Furthermore, in the Third National Health and Nutrition Examination Survey conducted between 1988 and 1994 in adults with T2DM and chronic kidney disease (e-GFR lower than 60 mL/min/1.73 m^2^), one third of patients did not have proteinuria, nor diabetic retinopathy, suggesting that this last group of patients might have suffered from a renal disease different from classical diabetic nephropathy [15]. Nephroangiosclerosis is a common finding in diabetic patients with renal insufficiency and absence of albuminuria [16,17].

## 3. Biopsy Indication

Renal biopsy is indicated in diabetic patients with an atypical presentation of renal disease that could be attributed to other renal entities different from diabetic nephropathy. Atypical presentation of renal disease in diabetic patients include microalbuminuria without diabetic retinopathy, rapid decline of glomerular filtration rate, rapid increase of proteinuria, sudden appearance of the nephrotic syndrome, active sediment or the appearance of signs and symptoms of systemic diseases (Table 1) [8,18].

**Table 1:** Atypical presentation of renal disease in diabetic patients.

Absence of diabetic retinopathy
Presence of hematuria
Active urinary sediment (acanthocytes, casts)
Less than 5 years of evolution of diabetes
Sudden onset of macroalbuminuria
Presentation as a nephrotic syndrome
Rapid decline of renal function
Acute renal failure
Clinical suspicion of other nephropathies: vasculitis, glomerulonephritis, amiloidoses
Markers of systemic diseases: Low complement, ANCA, ANA, dsDNA, cardiolipin antibody, ASLO, HIV, M-Spike in serum or urine, cryoglobulins, HBsAg, HCV
Significant reduction in the GFR (>30%) after ACE inhibitors or angiotensin II receptor blockers

ANA: antinuclear antibody; dsDNA: double-strand DNA; ASLO: anti-streptolysin O; ANCA: antineutrophil cytoplasmic antibody; HBsAg: Hepatitis B surface antigen; HCV: Hepatitis C virus; HIV: human immunodeficiency virus.

Early diagnosis of diseases different from diabetic nephropathy in diabetic patients is fundamental to preserve renal function in patients with renal diseases for which the natural history can be modified by treatment, especially in primary glomerulonephritis, systemic diseases with renal involvement or interstitial nephritis.

## 4. Histological Classification of Diabetic Nephropathy

The International Consensus Document for the Classification of T1DM and T2DM diabetes was published in 2010 [9]. The first consensus document for the classification of renal pathology was published in 1993 as a proposal for grading renal damage and classifying kidney disease in renal allograft biopsies, the so called Banff criteria [19]. A decade later, other international consensus for the classification of IgA nephropathy [20], lupus nephritis [21] or focal segmental glomerulosclerosis [22] were published. These documents provide a common language for pathologists, nephrologists, surgeons and basic scientists, and propose a grading and classification system that can be tested and fine-tuned as new knowledge appears. The utility of any classification proposed by consensus depends on its reproducibility and the accuracy to predict outcome. The oldest consensus classification, The Banff Classification, has been modified several times, but has clearly contributed to speed up knowledge and to improve the accuracy of the diagnosis of the different conditions affecting the renal allograft [23,24].

The consensus document for the evaluation of diabetic nephropathy establishes a minimum standard of quality to evaluate biopsies. Minimum sample size for adequate evaluation is 10 glomeruli and the biopsy should be stained with hematoxylin and eosin, periodic acid-Schiff (PAS), Masson trichrome, and periodic acid methenamine silver stains. Immunofluorescence requires the use of antibodies against IgA, IgG, IgM, C3, C1q, kappa and lambda light chains to rule out other renal diseases. A sample must be processed for electron microscopy.

The severity of lesions is graded according to glomerular involvement in four classes. Class I is characterized by normal optimal microscopy and basal glomerular thickening in electron microscopy, Class II is characterized by mesangial expansion and it is subdivided in Class IIa and Class IIb according to the severity of this lesion. Class III is characterized by the presence of at least one nodular lesion (Kimmelstiel-Wilson lesion), provided that there are no more than 50% of sclerosed glomeruli and Class IV or advanced diabetic glomerulosclerosis designates biopsies with more than 50% glomerulosclerosis when this lesion can be attributed to diabetic nephropathy, that is, the presence of Class II or III lesions or a long history of diabetes and diabetic retinopathy. Additionally, the degree of interstitial fibrosis, interstitial inflammation, arteriolar hyalinosis and arteriosclerosis are graded. However, these findings are not taken into consideration for the final grading of diabetic nephropathy. Coexisting disorders have to be also reported.

Interstitial fibrosis and tubular atrophy (IFTA) is evaluated with a 0–3+ semiquantitative scale as the percentage of the total involved area of interstitium and tubules as follows: 0 for absence of interstitial fibrosis, 1 for <25%, 2 for 25%–50% and 3 for >50%. In this classification system, the threshold between 0 and 1 is different from the Banff criteria that considers <10% of interstitial fibrosis as normal. This strict criteria to define a normal tubulo-interstitium will necessarily imply that a large proportion of patients have a pathological tubulo-interstitial structure. Interstitial inflammation is graded with a 0–2+ semiquantitative scale, 0 for absence of inflammation, 1 for inflammation in areas of IFTA and 2 for inflammation in areas with a normal tubulo-interstitial structure. Arterial hyalinosis is also classified with a 0–2+ semiquantitative scale as 0 for absent, 1 for at least one area of hyaline changes and 2 for more than one area of arteriolar hyalinosis. Finally, it should be reported whether the biopsy contains large arteries (yes/no) and arteriosclerosis is graded in the most affected artery as 0 for no intimal thickening, 1 when intimal thickening is less than the thickness of the media and 2 when intimal thickening is wider than the media. This proposal represents a very important step forward, since it can be viewed as a starting point that will allow further understanding of the meaning of diabetic lesions as the utility of the classification is evaluated in different clinical settings [25].

## 5. Reproducibility of Glomerular Lesions

The utility of any pathological classification depends on the degree of intra and interobserver reproducibility and its capacity for risk stratification. Interobserver reproducibility was evaluated by the authors of the consensus document. For this purpose, five observers evaluated 25 biopsies with diabetic nephropathy into class I, II, III and IV and the results of this pilot study suggest a satisfactory interobserver reproducibility for diabetic glomerular lesions (intraclass correlation coefficient of 0.84) [9].

## 6. Histological Damage and Outcome

Different studies have evaluated the utility of The International Consensus Document for the Classification T1DM and T2DM to predict risk for renal function deterioration and end stage renal disease. In a study including 50 patients with biopsy proven pure diabetic nephropathy, five year renal survival rates were associated with diabetic nephropathy class [26]. In another study published the same year, including 69 T2DM patients with biopsy proven diabetic nephropathy who did not present any class I cases, interstitial fibrosis and tubular atrophy and the severity of interstitial inflammation, but not glomerular class, were associated with the main outcome variable defined as initiation of dialysis or doubling of serum creatinine at the end of follow up [26]. Despite the small size of both studies, they suggest the importance of glomerular and tubulo-interstitial lesions as predictors of outcome in diabetic nephropathy.

Mise *et al.* [27] retrospectively reviewed 310 biopsies made in patients with diabetes between 1985 and 2010. In order to evaluate the relationship between biopsy findings and renal outcome, they discarded patients with coexistence of other diseases. Finally, they included 205 patients with the diagnosis of diabetic nephropathy. In their study, indications for renal biopsy were proteinuria >0.5 g/24 h, diabetes without diabetic retinopathy or the presence of hematuria. All biopsies were re-evaluated by one observer according to The International Consensus Document Guidelines. After correcting for confounding variables (age, gender, e-GFR, type of diabetes, urinary protein excretion, systolic blood pressure, body mass index, HbA1c, diabetic retinopathy and presence of red blood cells in the urinary sediment) they were able to show that hazard rate (HR) for end stage chronic renal failure, defined as the need for dialysis, increased with glomerular class. Class IIa was considered the reference value and HR and 95% confidence interval for glomerular classes I, IIb, III and IV were 0.21 (95% CI: 0.04–1.25), 2.12 (0.89–5.04), 4.23 (1.80–9.90), and 3.27 (1.32–8.10), respectively [27]. Other important findings were that degree of IFTA score, interstitial inflammation score, arteriolar hyalinosis and arteriosclerosis score correlated with the main outcome variable. The risk for end stage renal disease increased as damage score increases for IFTA, arteriolar hyaline changes and intimal thickening. Both inflammation in areas of IFTA (score 1) and in healthy areas (score 2) were associated with a significant risk for end stage chronic renal failure suggesting that even mild tubulo-interstital inflammation is an important determinant of outcome in diabetic nephropathy. This study confirms the utility of the International Consensus Document to classify the risk for progression to end stage renal disease. However, in the present study it was not analysed which of the evaluated lesions were independent predictors of outcome from glomerular lesions. In another retrospective study performed between 2003 and 2011 [28,29] including 396 patients with T2DM with biopsy proven diabetic nephropathy, the utility of histology to predict the risk for end-stage renal disease or doubling of serum creatinine was evaluated. Renal biopsy was indicated in patients with persistent albuminuria, decreased serum creatinine, sudden onset of proteinuria, hematuria or rapid progression of renal insufficiency. After five years of follow-up, renal survival rates were 100% in class I diabetic nephropathy, 90.1% in class IIa, 75.4% in class IIb, 39.0% in class III and 15.1% in class IV. After adjusting for baseline mean arterial blood pressure, proteinuria and e-GFR, glomerular lesions remained as an independent risk factor for progression to end stage renal disease and for doubling of serum creatinine. IFTA and interstitial inflammation were associated with renal outcome in the univariate analysis, however, only IFTA remained an independent predictor of outcome once the statistical model was adjusted for proteinuria, mean arterial blood pressure and e-GFR rate, further suggesting that apart from glomerular class, tubulo-interstitial burden of injury is an independent predictor of outcome. In this regard, the rate of decline of e-GFR was evaluated in patients with T2DM and macroalbuminuria that were biopsied and showed pure diabetic nephropathy according to the International Consensus Document. In this study, proteinuria and the degree of IFTA, but not glomerular class, were independent predictors of outcome. These data further suggest the importance of tubulointerstitial damage as a predictor of outcome [30].

Characteristically, biopsies from patients with diabetic nephropathy show a linear immunofluorescent staining for immunoglobulin G (IgG) along the glomerular and tubular basement membranes. These deposits are not due to immune-complex deposition but to non-specific trapping of immunoglobulins. The predictive value of the intensity of IgG immunofluorescence has been evaluated in a study including 165 patients with class I to III diabetic nephropathy. Biopsies were classified according to immunofluorescence intensity in three categories: 0 for absence of immunofluorescence, 1 for weak and 2 for intense staining. The main outcome variable was end stage renal disease. After adjusting for clinical and histological variables, the HR for end stage renal disease was 3.01 (1.05–8.68) for patients with weak and 4.68 (1.67–13.1) for patients with intense IgG immunofluorescence staining. Despite the fact that there was a weak association between glomerular class or glomerular basement membrane thickness and immunofluorescence intensity, the predictive value of IgG linear staining was independent from histological parameters. This study suggested that the intensity of linear staining is related to the severity of basement membrane involvement in diabetes. However, reproducibility of immunofluorescence intensity has to be evaluated and the potential utility of this observation should be confirmed in an independent cohort [31].

## 7. Renal Biopsy in Clinical Trials Aimed to Modify the Natural History of Diabetic Nephropathy

In the last years, a large number of new drugs with the potential for slowing renal function decline in diabetic patients have been tested in different clinical trials such as Bardoxolone [32], Pyridorin [33], Pentoxyfilin [34] and others [35].

Clinical trial design in diabetic nephropathy is aimed to show benefit of a new drug on top of renin angiotensin system blockade. Inclusion criteria are based on clinical parameters such as renal function and degree of proteinuria. Unfortunately, a proportion of patients with non-diabetic nephropathy and patients with diabetic nephropathy associated with other renal diseases [10] will be necessarily included in trials selecting patients according to clinical criteria. Accordingly, only kidney biopsy at entry may allow to exclude patients with renal diseases different from diabetic nephropathy or to stratify patients according to histological diagnosis.

The severity of chronic lesions in diabetic nephropathy roughly correlates with degree of proteinuria and renal functional impairment. Inclusion of patients with macroalbuminuria and severe renal functional impairment may imply that the severity of lesions may be already beyond the threshold of reversibility in a high proportion of patients. Theoretically, the maximal beneficial effect of new anti-inflammatory drugs should be obtained in patients with inflammation and relatively preserved renal structure. Thus, stratification for the severity of histological damage and severity of renal inflammation may be helpful to better understand which patients will obtain the maximal benefit of a new drug. However, the recruitment of a sufficient number of patients for the inclusion of a renal biopsy to include and stratify patients in diabetic nephropathy trials may be difficult.

The Renin-Angiotensin Study (RASS) was a five-year multicenter, randomized, double-blind, placebo-controlled trial comparing enalapril and losartan with placebo on early renal structural changes from diabetic nephropathy in T1DM patients without hypertension, albumin excretion rate above 20 mg/min and a glomerular filtration over 90/mL/min/1.73 m^2^. The primary study end point was a change in the mesangial volume fraction evaluated by means of a renal biopsy performed at entry and at five years of follow up. Secondary renal end points included changes in other glomerular, vascular, tubular, and interstitial variables and changes in the albumin excretion rate and renal function. In this study it was not possible to show any beneficial effect of treatment in comparison to placebo in the progression of mesangial volume fraction at this very early stage of disease. However, this study raises the question whether renal biopsy may constitute a useful tool to better characterize the effect of new drugs to modify the natural history of diabetic nephropathy [36]. In the same cohort of patients, macrophage protein 1 (MCP1)/creatinine ratio was measured at entry. This surrogate of renal inflammation was associated with an increased interstitial volume fraction at five years in females but not in males, pointing out that early inflammation may constitute a risk factor for the progression of histological damage in diabetic patients [36].

## 8. Conclusions

Only a proportion of diabetic patients with renal impairment are biopsied. Thus the preferred term for renal disease in diabetics is diabetic kidney disease, while the term diabetic nephropathy is only employed in cases with histological confirmation. Available data suggest that approximately one third of patients with diabetes that are biopsied show diabetic nephropathy, another third diabetic nephropathy with a superimposed non-diabetic disease and the other third a non-diabetic condition. The International Consensus Document for the Classification of T1DM and T2DM constitutes an important step forward to provide the international community with a common language for the classification of diabetic nephropathy. Its clinical utility has already been confirmed in different retrospective studies. New treatments for diabetic kidney disease are being tested in different clinical trials. The potential utility of renal biopsy to improve the design of clinical trials aimed to modify the natural history of diabetic nephropathy remains a matter of discussion.
